# Stathmin 1 is a biomarker for diagnosis of microvascular invasion to predict prognosis of early hepatocellular carcinoma

**DOI:** 10.1038/s41419-022-04625-y

**Published:** 2022-02-24

**Authors:** Yongchao Cai, Yong Fu, Changcheng Liu, Xicheng Wang, Pu You, Xiuhua Li, Yanxiang Song, Xiaolan Mu, Ting Fang, Yang Yang, Yuying Gu, Haibin Zhang, Zhiying He

**Affiliations:** 1 Institute for Regenerative Medicine, Shanghai East Hospital, School of Life Sciences and Technology, Tongji University School of Medicine, Shanghai, 200123 P. R. China; 2 Shanghai Engineering Research Center of Stem Cells Translational Medicine, Shanghai, 200335 P. R. China; 3 Shanghai Institute of Stem Cell Research and Clinical Translation, Shanghai, 200120 P. R. China; 4 Department of Liver Surgery V, Shanghai Eastern Hepatobiliary Surgery Hospital, Shanghai, 200438 P. R. China; 5 Institute of Brain-Intelligence Science and Technology, Zhangjiang Laboratory & Shanghai Research Center for Brain Science and Brain-Inspired Intelligence, Shanghai, 201210 P. R. China; 6 Department of cardiology, Shanghai East Hospital, Tongji University School of Medicine, Shanghai, 200120 P.R. China

**Keywords:** Tumour biomarkers, Diagnostic markers

## Abstract

Microvascular invasion (MVI) is presently evaluated as a high-risk factor to be directly relative to postoperative prognosis of hepatocellular carcinoma (HCC). Up to now, diagnosis of MVI mainly depends on the postoperative pathological analyses with H&E staining assay, based on numbers and distribution characteristics of MVI to classify the risk levels of MVI. However, such pathological analyses lack the specificity to discriminate MVI in HCC specimens, especially in complicated pathological tissues. In addition, the efficiency to precisely define stages of MVI is not satisfied. Thus, any biomarker for both conforming diagnosis of MVI and staging its levels will efficiently and effectively promote the prediction of early postoperative recurrence and metastasis for HCC. Through bioinformatics analysis and clinical sample verification, we discovered that Stathmin 1 (STMN1) gene was significantly up-regulated at the locations of MVI. Combining STMN1 immunostaining with classic H&E staining assays, we established a new protocol for MVI pathological diagnosis. Next, we found that the degrees of MVI risk could be graded according to expression levels of STMN1 for prognosis prediction on recurrence rates and overall survival in early HCC patients. STMN1 affected epithelial-mesenchymal transformation (EMT) of HCC cells by regulating the dynamic balance of microtubules through signaling of “STMN1-Microtubule-EMT” axis. Inhibition of STMN1 expression in HCC cells reduced their lung metastatic ability in recipients of mouse model, suggesting that STMN1 also could be a potential therapeutic target for inhibiting HCC metastasis. Therefore, we conclude that STMN1 has potentials for clinical applications as a biomarker for both pathological diagnosis and prognostic prediction, as well as a therapeutic target for HCC.

## Introduction

Hepatocellular carcinoma (HCC) is one of the four most fatal cancers worldwide [1]. Only 5–10% of HCC patients can be treated through surgery, and over 70% of patients have recurred in 5 years after surgery, including two-thirds of them recurred within 2 years [2, 3]. Vascular invasion (VI) is recognized as a strong risk factor leading to early recurrence and poor survival following curative resection in HCC, which is generally detected under macroscopic or microscopic observations for the diagnosis of HCC [4]. Compared to macroscopic VI or portal vein tumor thrombus (PVTT), microscopic VI (MVI) occurs at early stage of vascular invasion and metastasis of HCC, which carries the key pathological features found in early assessment of recurrence and metastasis for the risks of HCC patients [4]. Therefore, MVI is regarded as a key pathological factor leading to postoperative recurrence and metastasis of HCC, and a breakthrough point reflecting whether the postoperative survival of HCC patients will be improved.

Objective diagnosis to reveal the locations, amounts, and distribution characteristics of MVI is not only the essential element for pathological diagnosis of HCC, but also the key link in predicting prognosis and planning individualized therapeutic regimens for HCC patients [4–6]. Currently, multi-point sampling method with H&E staining on HCC specimens is mainly applied and viewed as a classic protocol for diagnosis of MVI. Risk levels of MVI are classified by the numbers and distribution characteristics of MVI [5, 7]. The risk levels of MVI were classified as M0: no MVI; M1 (low-risk group): ≤5 MVI in para-cancerous liver tissues; M2 (high-risk group): >5 MVI, or MVI in distant para-carcinoma liver tissue [5]. However, such strategy of diagnosis only based on H&E staining assay has a low specificity, especially when MVI locations are accompanied by inflammatory cell infiltration and tissue fibrosis or sclerosis [8]. In addition, differences in morphology and location of solitary tumors may lead to subjective error during multi-point samplings, while multiple-originated tumors may further result in additional uncertainties to correctly decide the sampling procedure [5, 7]. It was reported that the occurrence of MVI in specimens varied from 15% to 57.1% [8], which could be partially attributed to the discrepancy of diagnostic bias or the complexity of various influence factors of clinical pathology. Together, all of these adverse effects have influences on both accuracy of pathological diagnosis and precision on definition for staging of MVI, which further cause the difficulty in making accurate judgments on recurrence and metastasis of HCC patients finally. Therefore, it is necessary to find a biomarker for pathological diagnosis to clarify and stage MVI in HCC patients, in order to predict prognostic risks of recurrence and metastasis, and to plan the therapeutic regimens for recurrence and metastasis of HCC.

In present work, after exploring the HCC databases with PVTT or MVI features and then performing WGCNA algorithm, we successfully identified STMN1 as a candidate biomarker gene of MVI and STMN1 was closely correlative to HCC metastasis. We further discovered that STMN1 presented a unique expression pattern at MVI locations, and STMN1 expression varied in the HCC specimens from different patients. STMN1 was thoroughly studied as a biomarker of MVI to determine both the pathological diagnosis and risk classification of MVI. In addition, both in vitro and in vivo assays were used to confirm whether inhibition of STMN1 expression could reduce the metastasis capacity of HCC cells.

## Results

### STMN1 expression has close correlation with the occurrence of HCC metastasis

**Fig. 1:**
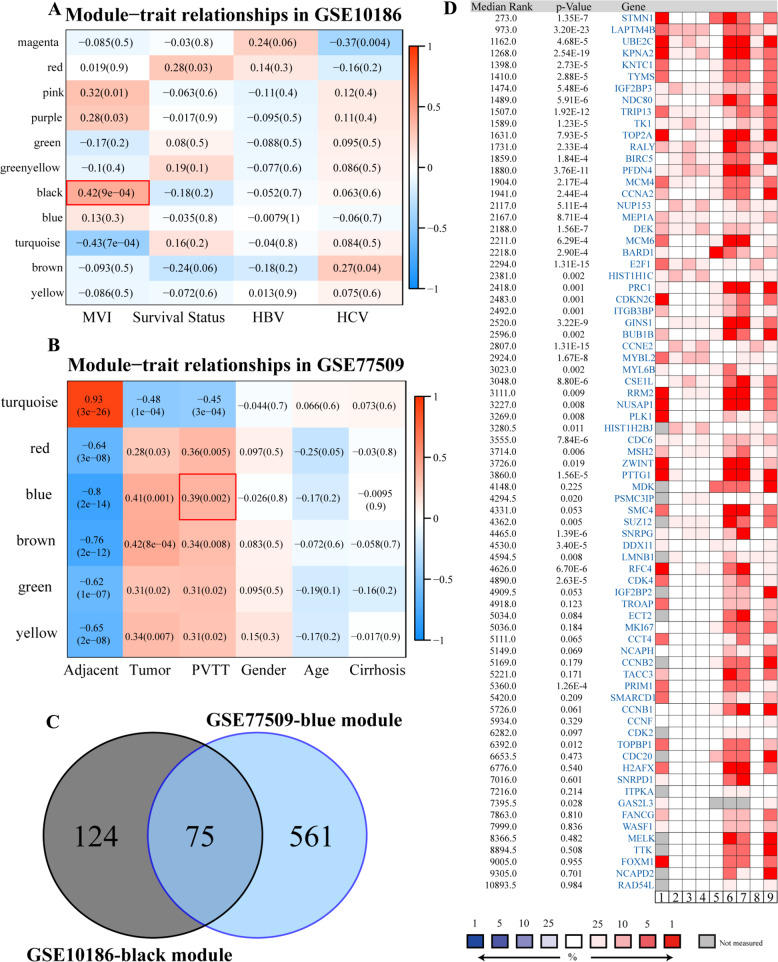
Exploration of potential biomarkers of vascular invasion. **A** WGCNA analysis of GSE10186. The module-trait relationships in GSE10186 were exhibited. “MVI” stands for HCC patients with microvascular invasion. **B** WGCNA analysis of GSE77509. The module-trait relationships in GSE77509 were presented. “PVTT” denotes HCC patients with Portal Vein Tumor Thrombosis. **C** Venn plot of black module in GSE10186 and blue module of GSE77509. **D** Meta-analysis of 75 overlapped genes across nine HCC datasets according to Oncomine database. The rank for 75 genes in the meta-analysis is the median rank across each of the differential analyses (tumor versus normal tissues). The *p*-value is calculated based on the median-ranked analysis. The related references of these HCC datasets were added in supplementary materials.

We started our study to search for the representative biomarkers that could recognize all levels of HCC metastasis in GEO databases. One of our study approaches was based on the known facts that MVI is a feature to judge early stage metastasis for HCC and that PVTT is used to evaluate metastasis at both middle and late stages. Two datasets, GSE10186 and GSE77509, were finally chosen for running WGCNA algorithm, which contained 17 HCC patients with MVI and 20 HCC patients with progressed to PVTT, respectively. Previously, WGCNA was successfully conducted to identify potential biomarkers or therapeutic targets according to the interconnectivity of gene sets and the linking between gene set and its phenotype [9]. From GSE10186, a well-composed module contained 199 genes to closely correlative to MVI phenotype (*r* = 0.42, *p* = 9e-04) (labeled in black color; Fig. 1A; Supplementary Fig. 1). On the other hand, from GSE77509, another module contained 636 genes to closely correlative to PVTT phenotype (*r* = 0.39, *p* = 0.002) (labeled in blue color; Fig. 1B; Supplementary Fig. 2). Next, Venn diagram was utilized to perform the intersection of the two modules from GSE10186 and GSE77509, respectively. Results revealed that there were 75 overlapped genes known from the intersection (Fig. 1C) and they might be correlative to both MVI and PVTT simultaneously.

In parallel, the Oncomine database was used to further explore the detailed correlations between 75 found genes and HCC. Oncomine is a classic online cancer microarray database that collected 4700 gene chips consisting of expression profiles for total of 480 × 10^6^ genes [10]. All nine HCC data in the Oncomine database were selected to perform a holistic meta-analysis, which were based on the differentially expressed features of these 75 genes (tumor versus normal tissues). The result showed that STMN1 was the most stably up-regulated gene in HCC tumor tissues among the total of 75 genes (*p* = 1.35e-7) (Fig. 1D).

### STMN1 has significantly increased in the invading cells at PVTT or MVI

**Fig. 2:**
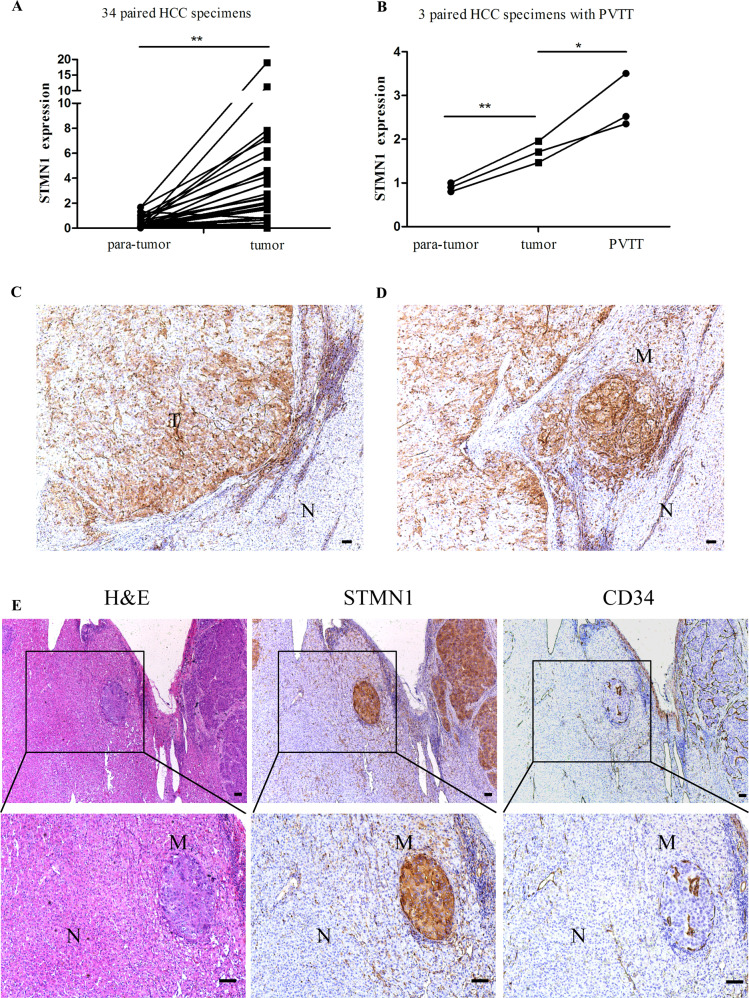
Expression and distribution of STMN1 in clinical HCC samples and tumor thrombus. **A** Levels of STMN1 mRNA in 34 HCC tissues and paired adjacent tissues. **B** Levels of STMN1 mRNA in three tumor thrombus, paired tumor, and adjacent tissues. **C**–**D** STMN1 protein levels and distribution were examined in HCC tissues, paired adjacent tissues, and microvascular invasion using immunohistochemistry. Scale bar: 100 um. **E** H&E and immunohistochemical staining of CD34 and STMN1 in HCC samples with MVI. Scale bar: 100 um.

To determine expression pattern of STMN1 in HCC specimens, we detected STMN1 expression in 34 HCC samples. Results of qPCR indicated that the STMN1 expression in HCC tissue were all significantly higher than those in the para-cancer tissue (Fig. 2A). In addition, para-tumor, tumor, and PVTT tissues of three advanced HCC patients with PVTT were collected for further study. Similarly, STMN1 expression showed the highest levels in the PVTT tissues. Remarkably, the significant differences in STMN1 expression levels were found between PVTT and regular tumor tissues, as well as between PVTT and para-tumor tissues (Fig. 2B).

Next, immunostaining assay indicated that STMN1 in tumor tissues was significantly higher than that in the matched para-tumor tissues while its expression levels at the site of tumor periphery was higher than that in tumor center (Fig. 2C). In addition, the expression levels of STMN1 in MVI tissues were significantly higher in regular tumor tissues (Fig. 2D). Usually, it has been known that MVI site is characterized by invasion of tumor cells into the blood vessels that were coated with CD34^+^ endothelial cells. In our pathological analyses with serial sections, H&E histological staining along with immunostainings of both STMN1 and CD34 were performed on the MVI-contained HCC specimens. The results clearly demonstrated that STMN1 specifically expressed at the site of MVI and the CD34^+^ vascular endothelial cells were around MVI with STMN1^+^ cells (Fig. 2E).

### STMN1 functions as a biomarker for the accurate pathological diagnosis of MVI

Based on the expression characteristics of STMN1 at site of MVI, we pursued to establish a new protocol to effectively and efficiently diagnose the MVI in clinical samples through using immunostaining of STMN1 in combination with H&E staining. During the process, a total of 130 cases of early HCC were especially collected. According to these traditional criteria [5], a total of 79 cases were diagnosed as MVI positive while 51 cases were diagnosed as MVI negative (M0). The risk level of MVI in 79 cases included that 42 cases were M1 and 37 cases were M2.

**Fig. 3:**
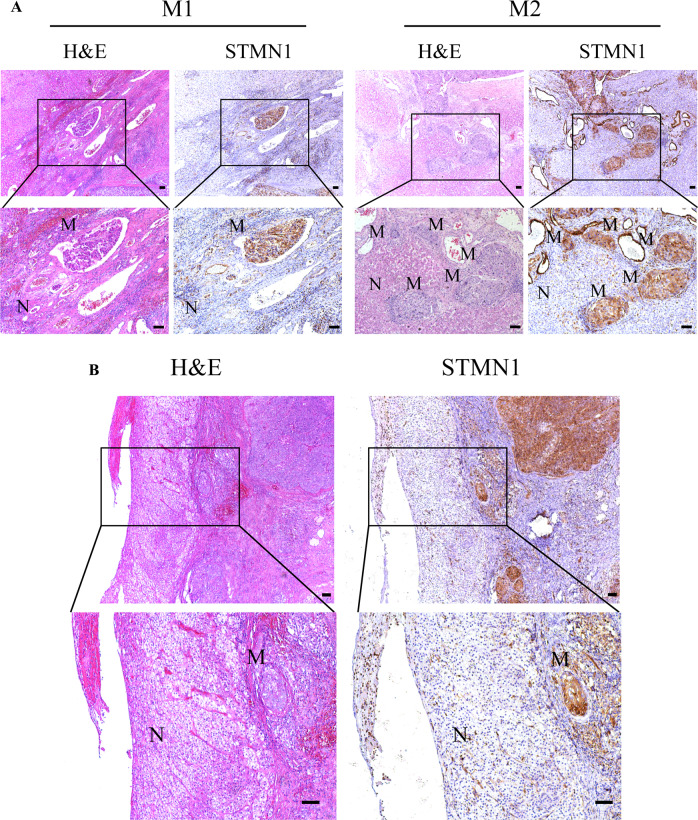
STMN1 is an MVI biomarker for diagnosis of early liver cancer specimens with MVI. **A** H&E and immunohistochemical staining of STMN1 in different rating of MVI (M1, M2). Scale bar: 100 um. **B** H&E and immunohistochemical staining of STMN1 in special samples with difficulties for pathological diagnosis of MVI. Scale bar: 100 um.

Remarkably, the results from our newly established method diagnosed with STMN1 immunostaining were all consistent with those of H&E staining for confirming MVI in 79 cases. However, our new method with STMN1 immunostaining was more efficient and effective than the classic diagnosis method (Fig. 3A). Additionally, our results also indicated that only based on H&E histological staining, it was difficult to successfully discriminate MVI in the HCC specimens from five cases, in which MVI was accompanied by other pathological features, such as inflammatory cell infiltration, severe tissue fibrosis, or sclerosis. Under these special conditions with difficulties for pathological diagnosis on HCC metastasis, STMN1 immunostaining had advantages to clearly discriminate MVI in the tissues with complex of pathological difficulties (Fig. 3B).

### Expression levels of STMN1 are correlative to the risk levels of MVI

**Fig. 4:**
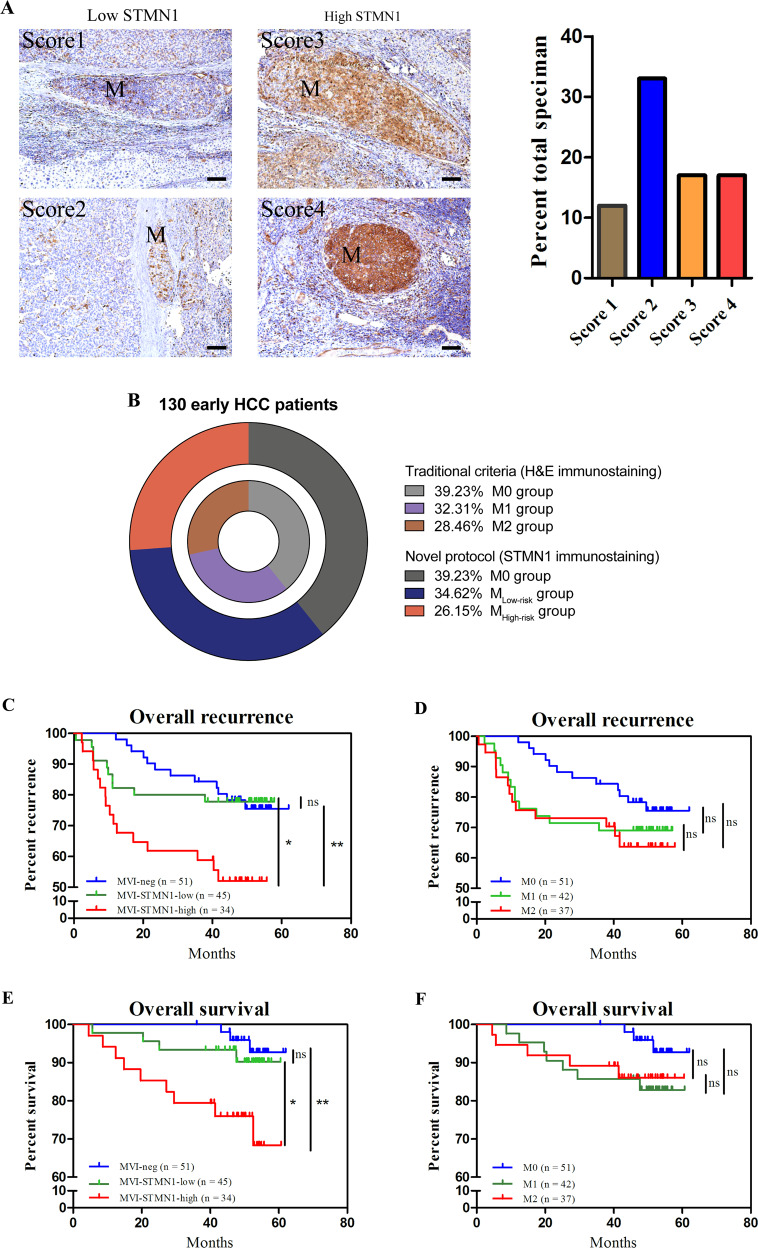
Expression of STMN1 in MVI is correlated with early recurrence. **A** HCC patients with MVI were classified into High-STMN1 group and Low-STMN1 group according to STMN1 protein levels and estimated scores in MVI tissues. Classification of 79 MVI samples based upon the IHC scores was also exhibited (right). Scale bar: 100 um. **B** Comparison between traditional criteria and our novel STMN1 protocol of classifying early HCC patients with MVI. **C**, **E** Kaplan–Meier curves of recurrence and overall survival (OS) displayed the correlation between clinical outcome and risk classification stratified by STMN1 in MVI. **D**, **F** Kaplan–Meier curves of recurrence and OS showed the correlation between clinical outcome and risk classification of MVI predicted by H&E staining.

Through the above results, we gradually realized that it might be applicable to diagnose the risk level of MVI based on precise expression levels of STMN1. Briefly, based on the results of STMN1 immunostaining on HCC specimens from 130 early HCC patients, the obtained expression levels of STMN1 were divided into three groups: (1) M0 group was defined as score of 0, based on detections with MVI negative (*n* = 51); (2) M_low-risk_ group was defined as score of 1–2, based on detections with MVI positive of low-STMN1 expression (*n* = 45); (3) and M_high-risk_ group was defined as score of 3–4, based on detections with MVI positive of high-STMN1 expression (*n* = 34) (Fig. 4A, B). Next, three scored levels of STMN1 expression were analyzed for their correlations with the clinic pathological parameters. Results revealed that the occurrence of MVI and tumor recurrence was mainly correlated to the grade of MVI (Table 1, *p* = 2.76E-23, *p* = 0.0288, respectively).

**Table 1:** Correlations between MVI stages and clinicopathologic features in early HCC patients.

Features	Total	M0	M_low-risk_	M_high-risk_	*p*-value
**Sex**					0.078
Male	103	43	37	23	
Female	27	8	8	11	
**Age**					0.616
<60	88	38	26	24	
≥60	42	13	19	10	
**HBsAg**					0.674
Negative	20	7	10	3	
Positive	110	44	35	31	
**Serum AFP**					0.002
<400	91	45	26	20	
≥400	39	6	19	14	
**Tumor size (cm)**					0.098
<2	24	12	9	3	
≥2	106	39	36	31	
**Cirrhosis**					0.542
Absent	87	35	31	21	
Present	43	16	14	13	
**Vascular invasion**					2.76E-23
Absent	51	51	0	0	
Present	79	0	45	34	
**Recurrence/ Metastasis**					0.0288
Absent	92	39	35	18	
Present	38	12	10	16	

Next, we also found that the recurrence rate was significantly higher in M_high-risk_ group than those in other two groups, respectively (Fig. 4C, *p* = 0.0237, *p* = 0.0053). More, Overall Survival (OS) in M_high-risk_ group was significantly lower than those in other two groups (Fig. 4E, *p* = 0.0317, *p* = 0.0029). In contrast, no significant difference in the recurrence rate and OS existed among M0, M1, and M2 groups diagnosed by H&E staining, the traditional diagnostic, and risk grading criteria for MVI (Fig. 4D, F).

### Correlation between STMN1 and HCC metastasis broadly exists in the clinical cases of HCC

**Fig. 5:**
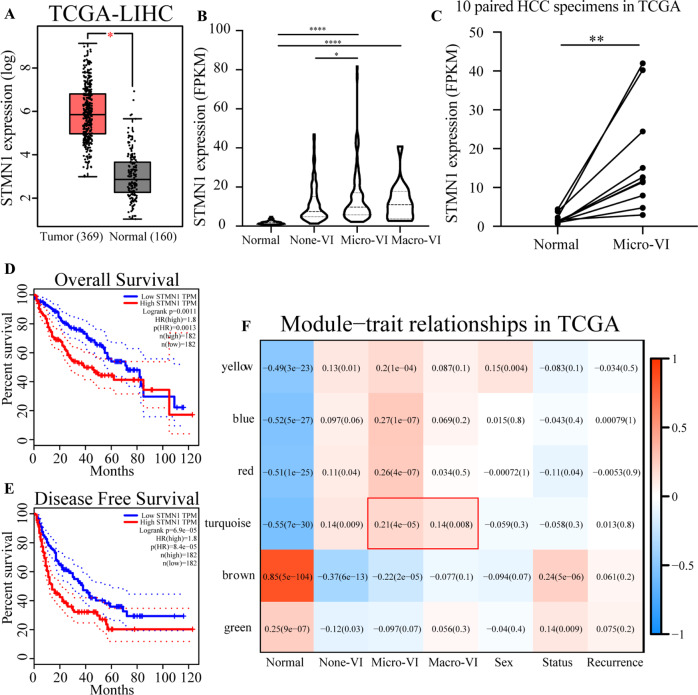
Oncogenic role of STMN1 in HCC validated by TCGA database. **A** Box plot of STMN1 expression between tumor and non-tumor tissues in HCC, analyzed by GEPIA database (http://gepia.cancer-pku.cn/) based upon TCGA data. **B** Violin plot of STMN1 expression according to different subgroups in TCGA database. **C** STMN1 expression in ten paired HCC specimens in TCGA database. **D**, **E** Overall Survival (OS) and Disease-Free Survival (DFS) of low-STMN1 group and high-STMN1 group in HCC. These results were also obtained from the GEPIA database. **F** WGCNA analysis of HCC patients in TCGA database. The detailed information on WGCNA was in Fig. S3. The module-trait relationships in TCGA were displayed. “None-VI”, “Micro-VI”, and “Macro-VI” means that HCC patients are with none vascular invasion, microvascular invasion (MVI), and macrovascular invasion, respectively.

To consolidate the correlation between STMN1 and tumor metastasis, we broadly analyzed the clinical cases of HCC through a screening on a large-scale of HCC specimens. Particularly, GEPIA [11], was adopted to analyze the expression pattern of STMN1 in HCC specimens. Results indicated that expression levels of STMN1 in HCC specimens were significantly higher than that in noncancerous tissues (Fig. 5A). More, STMN1 expression was reversely correlated to prognosis in terms of OS (*p* = 0.0011) and disease-free survival (DFS) (*p* = 6.9e-05) (Fig. 5D, E). Furthermore, the STMN1 expression in HCC specimens with MVI was all higher than those without MVI (Fig. 5B), revealing that the expression features of STMN1 from our small-scale HCC specimens were consistent with those from TCGA large-scale of HCC specimens. In addition, STMN1 was highly expressed in all tumor tissues from ten patients when compared to their matched non-tumor tissues collected from TCGA (Fig. 5C). Taken together, the distributions of STMN1 expression in TCGA HCC patients agreed with those results with small samples of HCC patients in our center (Fig. 2C–E).

Furthermore, similar to the results obtained from WGCNA algorithm on GSE10186 and GSE77509, the results from WGCNA on TCGA database also showed that STMN1 had closely correlations with both MVI and PVTT. The turquoise module was significantly relative to both MVI (*r* = 0.21, *p* = 4e-05) and PVTT (*r* = 0.14, *p* = 0.008), indicating that there was a close relationship between turquoise and metastasis (Fig. 5C, Supplementary Fig. S3). Notably, STMN1 was an essential component member of the turquoise module (*r* = 0.67, *p* = 4.17e-49), which further proved the existence of a special correlation between STMN1 up-regulation and tumor vascular invasion in HCC patients.

### STMN1 is involved in the activation of microtubular stability and EMT in vitro

**Fig. 6:**
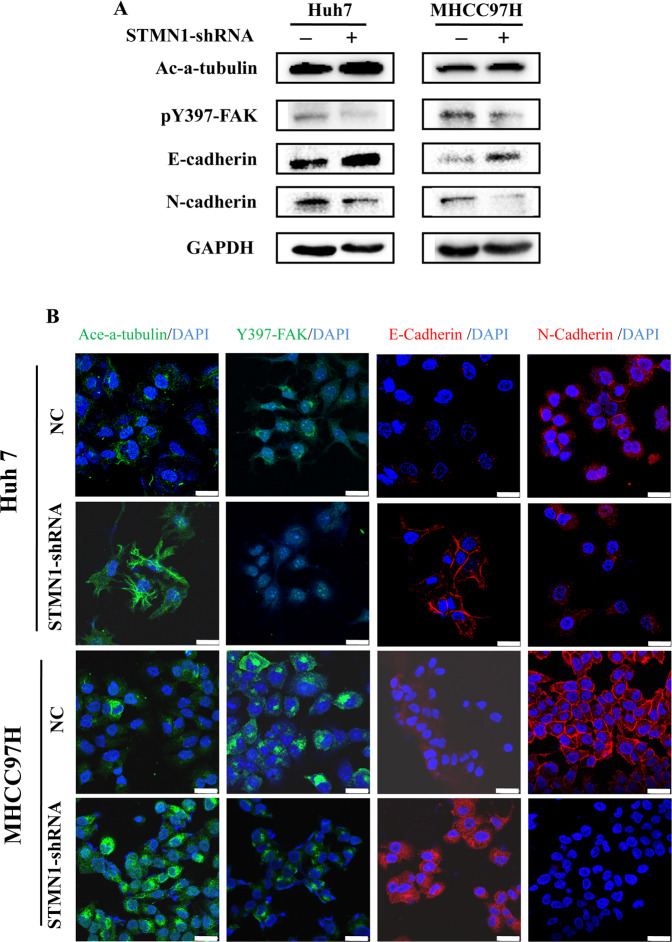
STMN1 knockout suppresses EMT via microtubule dynamics. **A** Validation of STMN1-Microtubule-EMT axis according to Western Blot in Huh7 and MHCC97H cells with STMN1 knockout. **B** Immunofluorescence assay revealed the existence of STMN1-Microtubule-EMT axis in Huh7 and MHCC97H cells. Representative confocal immunofluorescence images were displayed. Scale bar: 25 um.

Recent publications indicated that STMN1 promoted tumor invasion and metastasis through regulating the progress of EMT and the microtubule polymerization [12–15]. In similar, we also found that the expression of canonical EMT-related genes was related to STMN1 expression in pan-cancer analysis (Supplementary Fig. S4A) [16]. Specifically, STMN1 was positively correlated with mesenchymal markers while negatively associated with epithelial markers across 33 cancer types, including HCC (Supplementary Fig. S4A). To further confirm the changes of EMT process after STMN1 knockdown, we examined the expression levels of core EMT transcription factors and observed significant downregulation of Snail2 and ZEB1 in Huh7 and MHCC97H cells (Supplementary Fig. S4B–E). Other reports demonstrated that the microtubular stability inhibited EMT through the inhibition on activity of focal adhesion kinase (FAK) [17]. In parallel, we analyzed the activation of acetylated α-tubulin (a microtubular stability-specific and labeled protein) and the FAK specific labeled protein pY397-FAK in MHCC97H and Huh7 cells whose STMN1 were knocked down through shRNA lentivirus (Supplementary Fig. S5A–C). Results of western blot and immunostaining assays showed that expression level of acetylated α-tubulin increased while pY397-FAK decreased (Fig. 6A, B). These results suggested that STMN1 might promote EMT through regulating microtubular stability.

Moreover, results of western blot and immunostaining assays showed that N-Cadherin expression decreased while E-Cadherin increased in both Huh7 and MHCC97H cells after STMN1 knockdown, suggesting that EMT promoted by STMN1 activation could be affected by STMN1 knockdown (Fig. 6A, B). Our study suggested that the “STMN1-microtubule-EMT” axis existed in the progression of HCC.

### STMN1 knockdown reduces the cellular activities relative to HCC metastasis

**Fig. 7:**
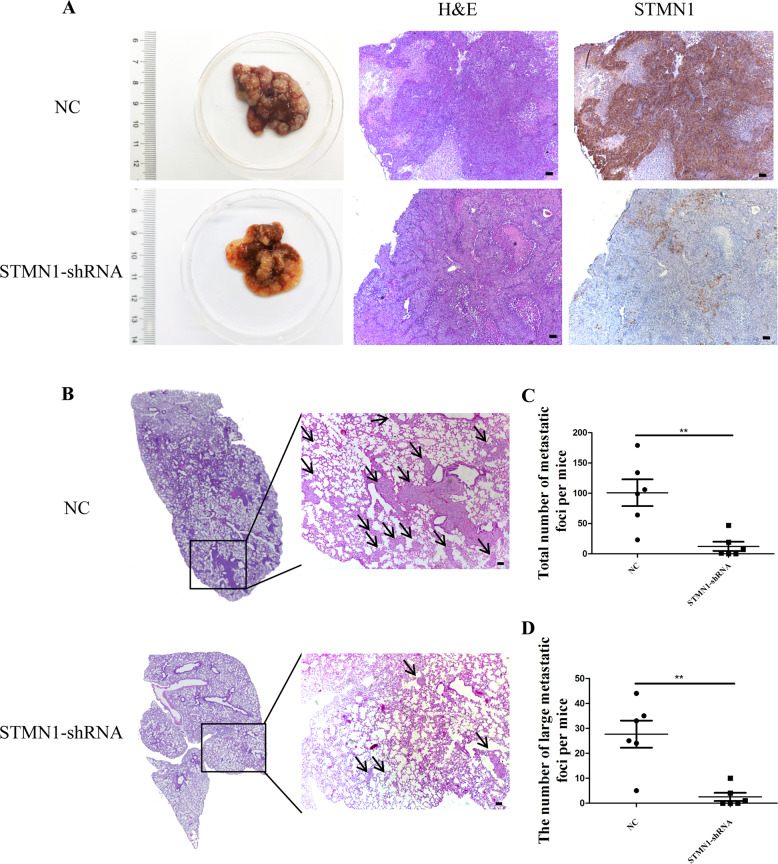
STMN1 knockdown inhibits the cellular activities relative to HCC metastasis in vivo. **A** Orthotopic tumor formation was performed in the mice recipients with spleen transplantation of HCC97H-NC/STMN1-shRNA cells. Representative images of excised liver samples were shown on the left. H&E and STMN1 staining of orthotopic tumors were shown on the right. Scale bar: 100 um. **B**–**D** In vivo experimental metastatic assay was performed via spleen transplantation with MHCC97H-shCtl/shSTMN1 cells. H&E staining of metastatic tumor in the lung, the total number of metastatic foci, and large metastatic foci per mice were exhibited. Scale bar: 100 um. **E** A vivid model explaining how STMN1 affected the formation of MVI in early HCC via mediating microtubular stability and EMT.

Given that STMN1 was involved in regulating both microtubular stability and EMT, the functions of STMN1 were further investigated during HCC metastasis in vivo. Here, result of wound healing and transwell invasion assays showed that STMN1 knockdown significantly inhibited HCC cell migration in vitro (Supplementary Fig. S5D–I). Previously, MHCC97H cells were known to have a highly metastatic malignant feature [18], The NOD-SCID-IL2R-gammaC-null (NSG) mice were transplanted with MHCC97H cells that were stably infected with either shRNA lentivirus interfered STMN1 or control negative lentivirus virus. Results showed that both the size and number of tumors in the interference group were smaller than those of the control group 4–6 weeks after transplantation, and the lung metastasis capacity of the HCC cells in the group transplanted with STMN1 interfered cells was also obviously inhibited in terms of both size and number of tumors (Fig. 7A–D). Thus, both in vitro and in vivo experiments demonstrated that STMN1 knockdown could inhibit HCC metastasis.

## Discussion

Finding specific markers to identify MVI is one of the practical strategies to solve the current difficulties of diagnosing MVI and thereby predicting the prognosis of early HCC [8, 19]. In this study, we directly find the clues from databases including HCC patients with macro- or micro-vascular invasion. Through WGCNA, we found that STMN1 was closely related to HCC. And the STMN1-diagnosed MVI was wrapped in CD34^+^ vascular endothelial cells, indicating that STMN1 could be used as a specific marker to diagnose MVI.

Usually, the risk grades of MVI should be used to accurately predict the prognosis of HCC patients. The higher the risk level of MVI, the poorer the disease-specific survival and recurrence-free survival in HCC patients [19, 20]. Roayaie et al. divided early HCC into non-, low-, medium- and high-risk MVI as an MVI grade from low to high according to the numbers of MVI under microscopy diagnoses based upon the distance from MVI to tumor [20]. Shuji Sumie et al. divided MVI into mild (1–5 MVIs) and severe MVI ( > 5 MVIs) according to the number of MVI invading vessels [19]. The DFS and OS of HCC patients in the MVI group were significantly higher than those in the non-MVI group. However, no statistical difference for both DFS and OS existed between low- and high-risk MVI groups. Remarkably, the contradiction between MVI grading and prognosis in different studies as mentioned above reflected the actual problem for the accuracy of MVI diagnosis. In our study, we demonstrated that the risk grades of MVI could be determined based on different expression levels of STMN1 in MVI. Thus, the patients can be classified into three groups (M0, M_low-risk_, and M_high-risk_). The recurrence rates were significantly different between MVI patients with low- and high-expression of STMN1, while both recurrences and survival rates were similar for both MVI patients with low expression of STMN1 and those without MVI. Besides, to make the comparisons among STMN1 and reported biomarkers, which can be used to predict the HCC prognosis or recurrence, including FN1, S100P, TSPAN12, MYC, WNK2, CD44, STIP1 [21–25], we analyzed the early stage HCC data in the TCGA and the uni- and multivariate cox regression analyses were both performed. The results showed that only STMN1 was an independent risk factor for early HCC recurrence (Supplementary Fig. S6A, B), and could affect recurrence rate of early HCC (Supplementary Fig. S6C). Interestingly, the ROC curve analysis indicated that none of these markers could predict the 3-year and 5-year recurrence rate of early HCC and only STMN1 could better predict the 1-year recurrence rate of early HCC (Supplementary Fig. S6D). This may be because other biomarkers are primarily studied for all stages of HCC, and some are primarily emphasized as serum markers. Taken together, STMN1 might be better than other molecules in predicting the recurrence of early HCC. Actually, literatures also reported that STMN1 could function as a secreted protein in serum and urine in various malignancies [26–28]. However, our preliminary results did not show significant differences, probably due to that the STMN1 secretion level was all beyond the limited sensitivity of the present detection method (data not shown). This study will be tried in future when the sensitivity of assay is improved, which is a research hotspot in the field of MVI in HCC.

In recent years, studies on some solid tumors have revealed that STMN1 is an independent risk factor for poor prognosis, local invasion, lymphatic metastasis, and chemotherapy resistance [29–32]. Consistent with these studies, the pan-analysis of STMN1 shows that STMN1 expressed differentially in most cancer types (Supplementary Fig. S7A, B). STMN1 was also a high-risk factor in many malignancies according to the univariate cox analysis across 33 cancer types. Intriguingly, no matter what endpoint events were considered in survival analyses, involving OS, DSS, DFI, and PFI, high-STMN1 expression tend to lead to poorer prognosis in HCC patients (Supplementary Fig. S7C). Additionally, the molecular mechanism by which STMN1 promotes HCC metastasis is unclear. Here, pan-cancer analysis provides strong hints between STMN1 and EMT, and in vitro experiments confirmed that STMN1 inhibition can increase the stability of microtubules and decrease the activity of adhesion spot kinase, thereby inhibiting the occurrence of EMT and affecting tumor metastasis (Fig. 7E).

In summary, the STMN1-immunostaining-contained new method is easier and more accurate for the diagnosis of MVI and the estimation of HCC prognosis, and therefore has an important clinical application value in diagnosing the risk level of MVI in patients with early HCC.

## Material and methods

### Human HCC tissues

A total of 130 early HCC samples with MVI and 34 HCC samples and their corresponding non-tumor tissues were obtained from HCC patients undergoing surgical resection at the Eastern Hepatobiliary Surgery Hospital (Shanghai, China) between 2016 and 2017. During the process, a total of 130 cases of early HCC were especially collected from the patients with solitary HCC (<5 cm) who underwent curative resection with surgical margin >1 cm. Patients without a margin of non-tumor tissue ≥1 cm were excluded. Different sites of HCC tissues according to 7-point baseline sampling protocol were examined microscopically by two senior pathologists to diagnose HCC and MVI independently. The clinical characteristics of 130 early HCC samples are listed in Supplementary Table 1. Written informed consent was obtained from all patients on the use of clinical samples for medical research. All human samples used in this study were approved by the Ethics Committee of Tongji University (Shanghai, China).

### Online data sources

To search for the key genes in metastasis of HCC, we obtained gene expression data from Gene Expression Omnibus (GEO) database (https://www.ncbi.nlm.nih.gov/gds/), including GSE10186 and GSE77509. The two datasets both had a part of HCC patients with obvious metastasis, either Portal Vein Tumor Thrombosis (PVTT) or microvascular invasion (MVI). On the other hand, we also gathered RNA-seq data from The Cancer Genome Atlas (TCGA) database (http://cancergenome.nih.gov). The detailed information was in Supplementary Table 2. To better understand the underlying function of hub genes, Oncomine database (https://software.oncomine.com) and GEPIA database (http://gepia.cancer-pku.cn/) were applied in our study. The nine analyzed datasets collected by Oncomine database from six research teams are as followed [33–38].

1. Chen Liver, MolBiol Cell, 2002; (PMID: 12058060).

2. Guichard Liver, Nat Genet, 2012; (PMID: 22561517).

3. Lamb Liver, PLoS One, 2011; (PMID: 21750698).

4. Mas Liver, Mol Med, 2008; (PMID: 19098997).

5. Roessler Liver, Cancer Res, 2010;(PMID: 21159642).

6. Wurmbach Liver, Hepatology, 2007. (PMID: 17393520).

### WGCNA analysis

We retrieved the RNA-seq data from GSE10186, GSE77509, and TCGA to conduct Weighted Gene Co-expression Network Analysis (WGCNA), respectively. The WGCNA R package was used to identify traits-related modules, especially macrovascular invasion and microvascular invasion (MVI). The related data was transformed into topological overlap matrix (TOM). Based on the TOM dissimilarity measure, Top 3000 differentially expressed genes from each data matrix, analyzed by the LIMMA R package (LIMMA version: 3.40.6), were assigned to a different module. In our study, we set soft-thresholding power as 18, scale-free R2 as >0.8, and minimal module size as 30 to figure out key modules. The modules of each dataset were then utilized to calculate their correlation with traits using Pearson’s correlation test and adjusted *p* < 0.05 was considered significant.

### Human hepatocellular carcinoma cell lines

The HCC cell line Huh7 and MHCC97H were obtained from the Shanghai Institute of Biochemistry and Cell Biology of the Chinese Academy of Sciences (Shanghai, China). All cell lines used in this study were routinely tested for mycoplasma contamination and authenticated by short tandem repeat analysis 6 months ago. The cells were cultured in Dulbecco’s Modified Eagle’s Medium (Gibco) containing 10% fetal bovine serum (Gibco) and 1% penicillin/streptomycin. All cell lines were incubated at 37 °C in a humidified incubator containing 5% CO2.

### RNA extraction and quantitative real-time PCR (qPCR) assay

Total RNA was extracted from HCC tissues or cells following the standard TRIZOL(Takara) protocol, and reverse transcription was synthesized using total RNA with a PrimeScript RT Master Mix (Takara) according to manufacturer’s instruction. Quantitative real-time PCR was performed using SYBR Green kit (Takara) on an ABI Prism Q7 system (Thermo Fisher Scientific, MA, USA) with the following primers: quantitative STMN1-forward: 5′-GATTGTGCAGAATACACTGCCTGT-3′; STMN1-reverse: 5′-TTGCGTCTTTCTTCTGCAGCTTCT- 3′; quantitative Snail2-forward: 5′-CGAACTGGACACACATACAGTG-3′; Snail2-reverse: 5′-CTGAGGATCTCTGGTTGTGGT-3′; quantitative ZEB1-forward: 5′-GATGATGAATGCGAGTCAGATGC-3′; ZEB1-reverse: 5′-ACAGCAGTGTCTTGTTGTTGT-3′. β-actin mRNA was used as the internal control.

### Western blot assay

Proteins were extracted using RIPA buffer (P0013B, Beyotime, Suzhou, China) supplemented with protease inhibitor cocktail (Roche), separated using sodium dodecylsulfate polyacrylamide gel electrophoresis (SDS-PAGE), and then electrophoretically transferred to a nitrocellulose membrane (HAHY00010, Millipore). The membrane was blocked in PBS-T containing 5% milk/BSA for 2 h before overnight incubation with a primary antibody at 4 °C. After 2 h incubation with a secondary antibody, signals were quantitated using an Odyssey infrared imaging system (LI-COR) at 700 nm or 800 nm. The information of primary antibodies was listed in Supplementary Table 3 and the original western blots are uploaded in Supplementary Fig. S8.

### Immunohistochemical assay

Paraffin-embedded HCC sections were obtained from the Eastern Hepatobiliary Surgery Hospital, Second Military Medical University, Shanghai, China. Tissue sections were deparaffinized using xylene and rehydrated in grade alcohols. After being treated for antigen retrieval at 121 °C for 2 min, the slides were treated with 3% H_2_O_2_ for 15 min. Then, sections were blocked in 5% BSA for 1 h at room temperature, incubated with proper primary antibodies overnight at 4 °C, and incubated with HRP-conjugated secondary antibodies at room temperature for 30 min, and the subsequent detection was performed using the standard substrate detection of HRP. The staining extent score was on a scale of 1–4, corresponding to the percentage of immunoreactive tumor cells and the staining intensity. IHC H-score was computed as the sum of 43 (4%) + 33 (3%) + 23 (2%) + 13 (1%).The detailed information of primary antibodies was listed in Supplementary Table 3.

### Immunofluorescent assay

Cells cultured in 12-well chamber slides were washed three times with PBS at room temperature, fixed with 4% paraformaldehyde for 15 min, permeabilized with 0.5% Triton X-100 for 20 min, blocked with 1% BSA, and incubated with a primary antibody overnight at 4 °C. After three times washes with PBS, the cells were incubated with a secondary antibody for 1 h at room temperature and washed three times with PBS. The cells were then mounted with DAPI for nuclear staining, and the images were acquired with an inverted confocal laser scanning microscope. The related information of primary antibodies was listed in Supplementary Table 3.

### Lentivirus-mediated STMN1 knockdown in cells

The recombinant lentivirus STMN1 shRNA was purchased from GENECHEM (Shanghai, China). Huh7 and MHCC97H cells were seeded in 6-well plates at 1 × 10^6^ cells per well and infected in the 50% confluence. Then, the stable cell lines were obtained by 1.5 ug/ml puromycin selection for 1 week. The efficiency of knockdown was examined by qPCR and western blot analysis.

### Cell migration and invasion in vitro

In vitro migration was performed using a wound healing scratch assay. Cells were plated in 6-well plates and cultured until confluent. Then the cells were drawn to make an artificial scratch and cultured after 72 h, the migration distance was measured.

In vitro invasion assays were conducted using transwell chambers with 8-um pore inserts (BD, Biosciences). The cells were seeded into the upper chambers, which were filled with serum-free medium. The lower chambers were filled with medium supplemented with 10% fetal bovine serum. After incubation for 48 h, the cells remaining on the upper membrane were removed with a cotton swab, and the chambers were fixed in 4% paraformaldehyde for 20 min, and stained with crystal violet.

### In vivo metastatic experiments in animal model

Six-week-old male NSG mice were obtained from Shanghai Model Organisms (Shanghai, China). To detect the metastatic ability of cells, 12 NSG mice were divided into two groups, orthotopic transplantation mouse model were established by spleen injecting 2 × 10^6^ MHCC97H cells. All of the mice were sacrificed after 4 weeks. Tumors, livers, and lungs excised from the mice were fixed in 4% paraformaldehyde and embedded in paraffin. Consecutive sections of those tissues were stained with hematoxylin and eosin. The number of lung metastases was calculated and evaluated independently.

### Statistical analyses

Statistical analyses were performed using SPSS software (18.0 version), and *p* < 0.05 was considered statistically significant. The Student *t*-test was used to analyze the data of experiments involving two groups. The Wilcoxon signed-rank test was used for the comparison of the expression levels of STMN1 in human HCC tissues and their adjacent noncancerous tissues. The Mann–Whitney *U*-test was used for comparison of tumor weight and volume of mice. The *χ*^2^-test was used to compare two sample rates. The survival curves were assessed using the Kaplan–Meier method, and statistical differences between the two groups were evaluated using a log-rank test.

## Supplementary information

Supplemental material

aj-checklist

## Data Availability

The authors declared that all the data and materials generated in this study are available from the corresponding author on reasonable request.
