# Supplementary material for: Stathmin 1 is a biomarker for diagnosis of microvascular invasion to predict prognosis of early hepatocellular carcinoma
Source: Cell Death Dis. 2022 Feb 24;13(2):176. doi: 10.1038/s41419-022-04625-y (PMC8873260; doi:10.1038/s41419-022-04625-y)
Supplement: Supplementary file 1 — Supplemental material [file 41419_2022_4625_MOESM1_ESM.docx]

Stathmin 1 is a biomarker for diagnosis of microvascular invasion to predict prognosis of early hepatocellular carcinoma

Yongchao Cai^1, 3, 4, 7^, Yong Fu^2, 7^, Changcheng Liu^1, 3, 4, 7^, Xicheng Wang^1, 3, 4, 7^, Pu You^5^, Xiuhua Li^1, 3, 4^, Yanxiang Song^1, 3, 4^, Xiaolan Mu^1, 3, 4^, Ting Fang^1, 3, 4^, Yang Yang^1, 3, 4^, Yuying Gu^6^, Haibin Zhang^2*^, Zhiying He^1, 3, 4*^

^1^Institute for Regenerative Medicine, Shanghai East Hospital, School of Life Sciences and Technology, Tongji University School of Medicine, Shanghai 200123, P. R. China.

^2^Department of Liver Surgery V, Shanghai Eastern Hepatobiliary Surgery Hospital, Shanghai 200438, P. R. China.

^3^Shanghai Engineering Research Center of Stem Cells Translational Medicine, Shanghai 200335, P. R. China.

^4^Shanghai Institute of Stem Cell Research and Clinical Translation, Shanghai 200120, P. R. China.

^5^Institute of Brain-Intelligence Science and Technology, Zhangjiang Laboratory & Shanghai Research Center for Brain Science and Brain-Inspired Intelligence, Shanghai 201210, P. R. China.

^6^Department of cardiology, Shanghai East Hospital, Tongji University School of Medicine, Shanghai 200120, P.R. China.

^7^These authors contributed equally: Yongchao Cai, Yong Fu, Changcheng Liu, Xicheng Wang.

E-mail: [drzhanghb@163.com](mailto:drzhanghb@163.com); [zyhe@tongji.edu.cn](mailto:zyhe@tongji.edu.cn).

## Supplementary Figures


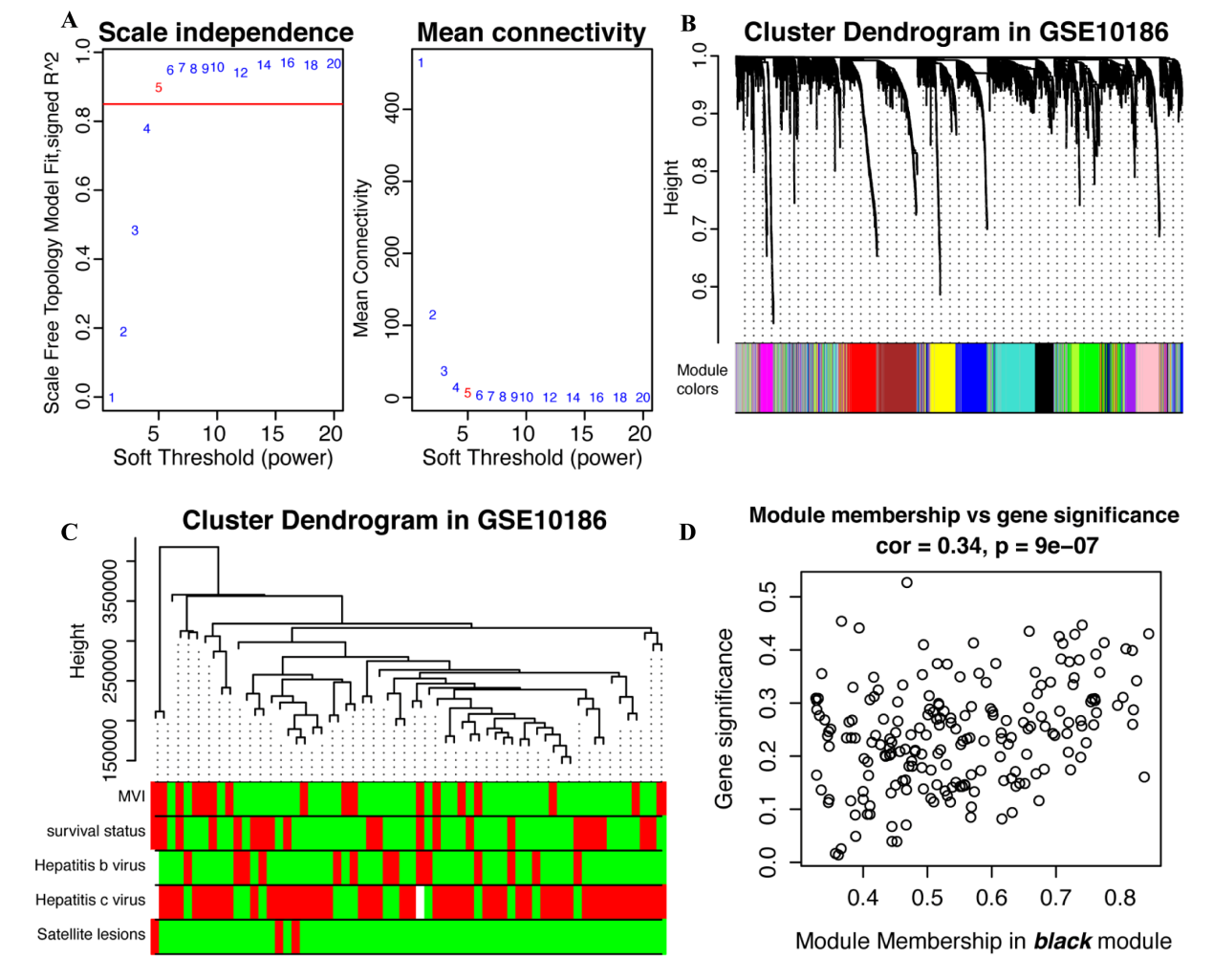


## Fig. S1. WGCNA analysis of GSE10186.

(A) Exploration of the scale-free topology fit (left) and the mean connectivity (right) for different soft-threshold (power). The best soft-thresholding power for GSE10186 was 5. (B) Cluster Dendrogram of GSE10186 based on a dissimilarity measure (1-TOM). (C) Cluster dendrogram of GSE10186. The clustering was based upon the GSE10186 mRNA data of differentially expressed genes from LIMMA analysis. Color intensity varies positively with MVI, survival status, Hepatitis B virus, Hepatitis C virus and satellite lesions. Red color means positive, green color denotes negative and white indicates related data missing. (D) Scatter plot of the correlation between black module and included genes.


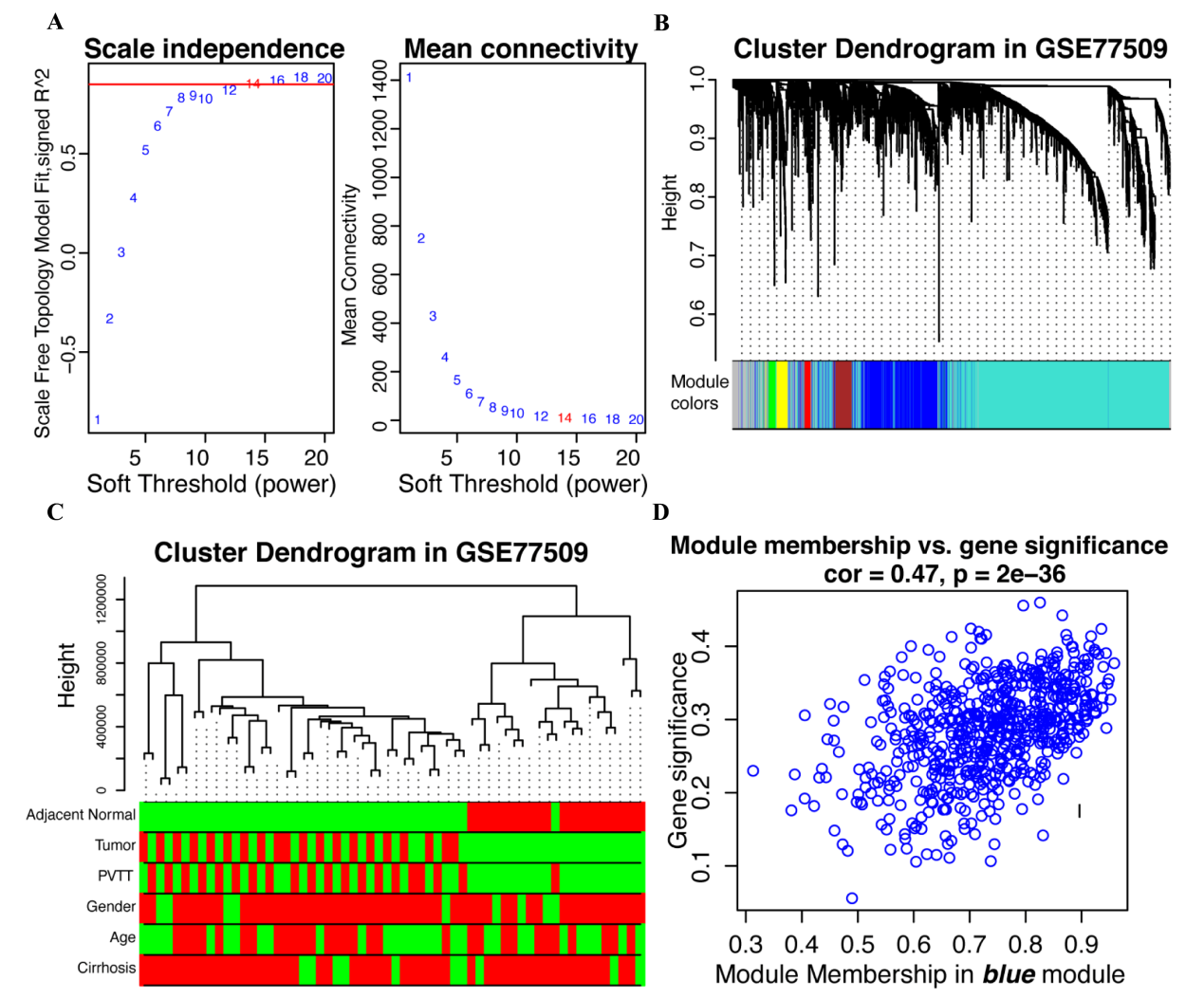


## Fig. S2. WGCNA analysis of GSE77509.

(A) Identification of the scale-free topology fit (left) and the mean connectivity (right) for multiple soft-threshold (power). The used soft-thresholding power for GSE77509 in our study was 14. (B) Cluster dendrogram of GSE77509 based on a dissimilarity measure (1-TOM). (C) Cluster dendrogram of GSE77509. The clustering was according to the GSE77509 mRNA data of differentially expressed genes from LIMMA analysis. Color intensity varies positively with adjacent normal tissues, tumor, PVTT, gender, age and cirrhosis. Red color stands for positive while green denotes negative. (D) Scatter plot of the correlation between blue module and involved genes.


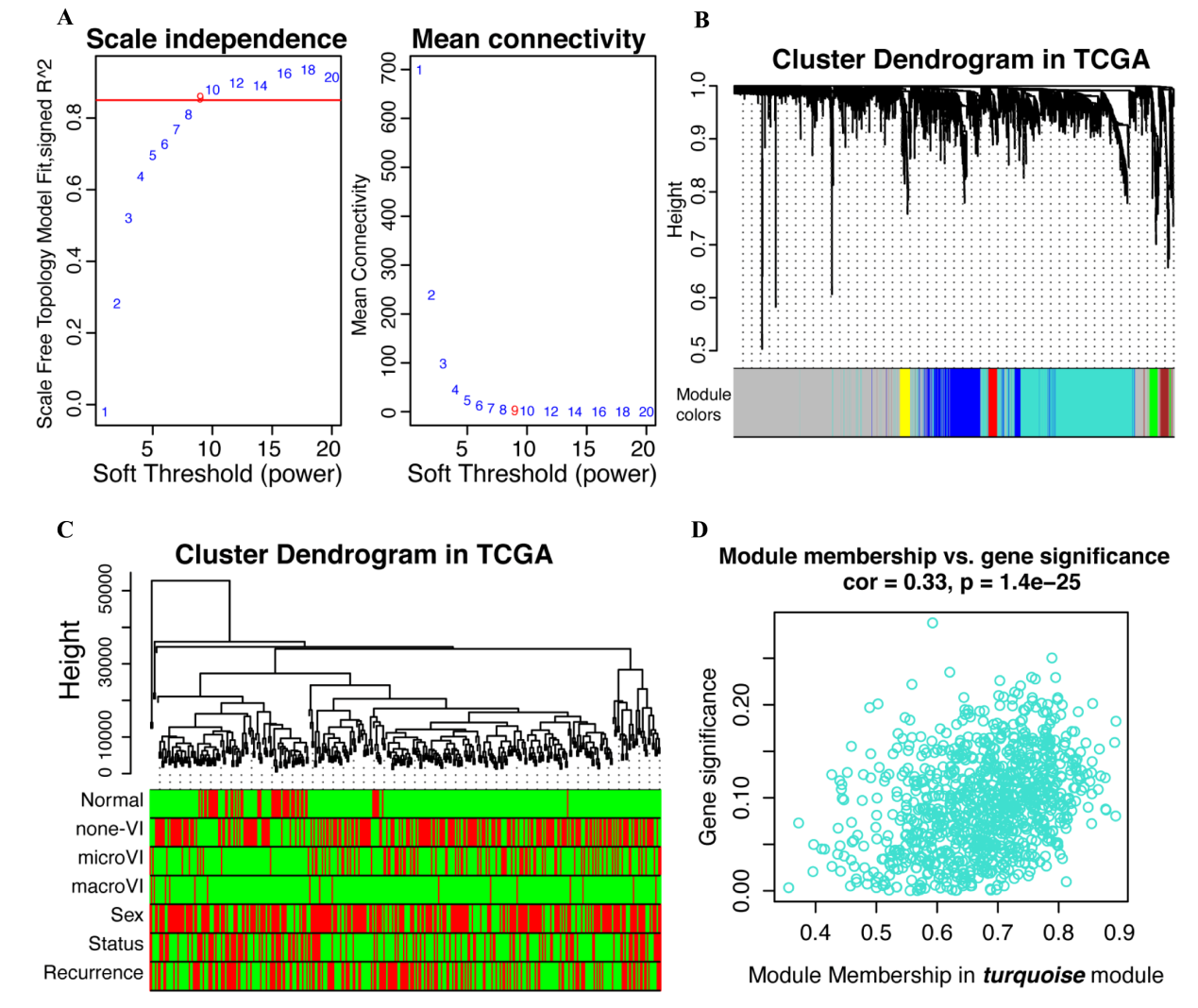


## Fig. S3. WGCNA analysis of TCGA-LIHC data.

(A) Investigation of the scale-free topology fit (left) and the mean connectivity (right) for several soft-threshold powers. The fit soft-thresholding power for GSE77509 in our study was 9. (B) Cluster dendrogram of TCGA-LIHC based on a dissimilarity measure (1-TOM). (C) Cluster dendrogram of TCGA-LIHC. The clustering was built on the TCGA-LIHC mRNA data of differentially expressed genes from LIMMA analysis. Color intensity varies positively with normal specimens, non-VI, micro-VI, macro-VI, sex, status and recurrence. Red color presents positive while green means negative. (D) Scatter plot of the correlation between turquoise module and contained genes


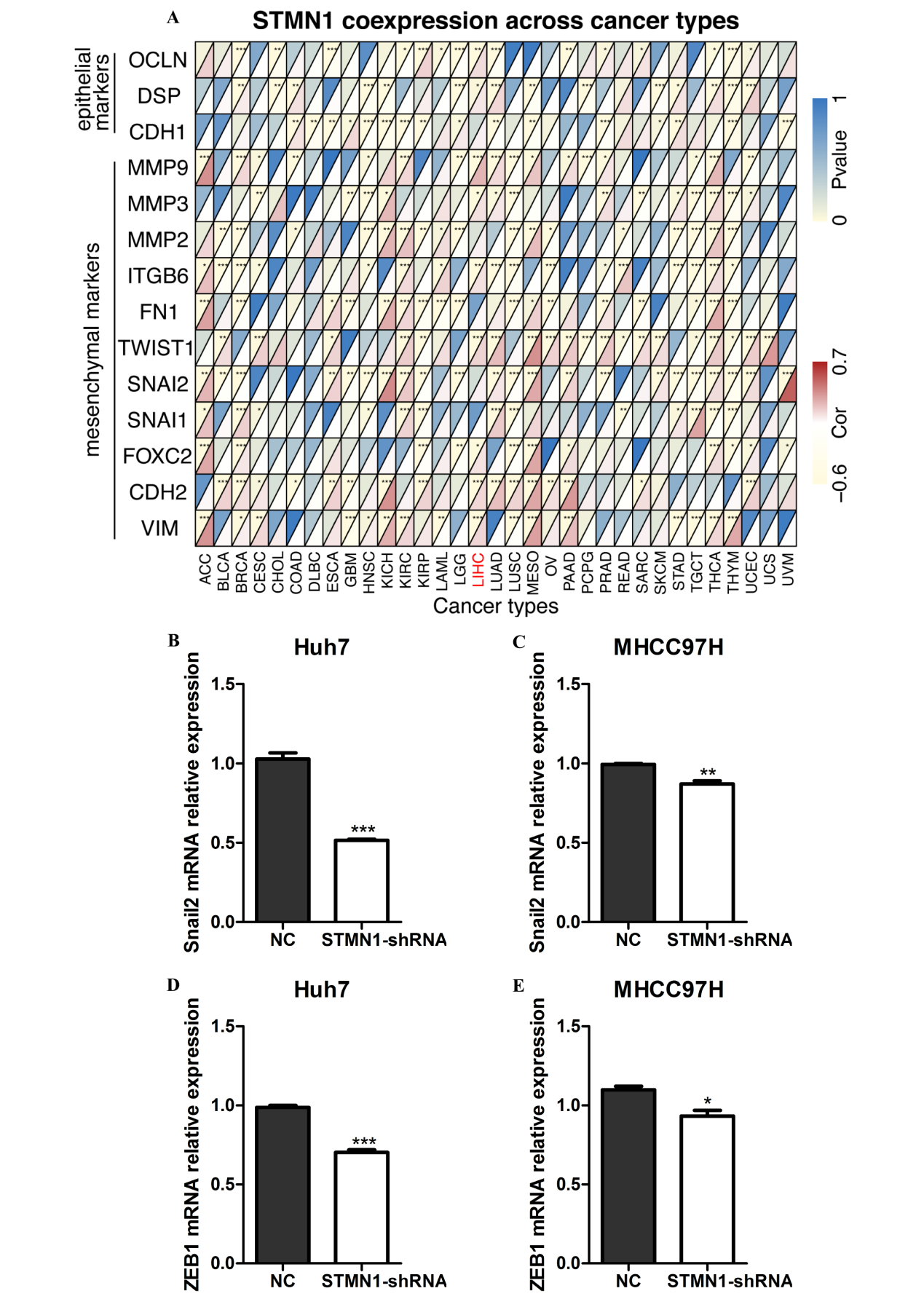


## Fig. S4. Correlation of STMN1 and EMT-related genes in TCGA database with coverage of 33 cancer types and expression levels of EMT transcription factors in Huh7 and MHCC97H after STMN1 knockdown

(A) The abbreviations used in the plot are as followed: ACC, adrenocortical carcinoma; BLCA, bladder urothelial carcinoma; BRCA, breast invasive carcinoma. CESC, cervical squamous cell carcinoma and endocervical adenocarcinoma; CHOL, cholangiocarcinoma; COAD, colon adenocarcinoma; COAD, READ, colon adenocarcinoma/rectum adenocarcinoma esophageal carcinoma; DLBC, lymphoid neoplasm diffuse large B-cell lymphoma; ESCA, esophageal carcinoma; GBM, glioblastoma; LGG, brain lower grade glioma; HNSC, head and neck squamous cell carcinoma; KICH, kidney chromophobe; KIRC, kidney renal clear cell carcinoma; KIRP, kidney renal papillary cell carcinoma; LAML, acute myeloid leukemia; LIHC, liver hepatocellular carcinoma; LUAD, lung adenocarcinoma; LUSC, lung squamous cell carcinoma; MESO, mesothelioma; OV, ovarian serous cystadenocarcinoma; PAAD, pancreatic adenocarcinoma; PCPG, pheochromocytoma and paraganglioma; PRAD, prostate adenocarcinoma; READ, rectum adenocarcinoma. SARC, sarcoma; SKCM, skin cutaneous melanoma; STAD, stomach adenocarcinoma; TGCT, testicular germ cell tumors; THCA, thyroid carcinoma; THYM, thymoma; UCEC, uterine corpus endometrial carcinoma. UCS, uterine carcinosarcoma; UVM, uveal melanoma. *, **, *** stands for *p* < 0.05, *p* < 0.01 and *p* < 0.001, respectively. (B, C) qRT-PCR analysis revealed downregulation of Snail2 in Huh7 and MHCC97H after STMN1 knockdown. (D, E) qRT-PCR analysis revealed downregulation of ZEB1 in Huh7 and MHCC97H after STMN1 knockdown.


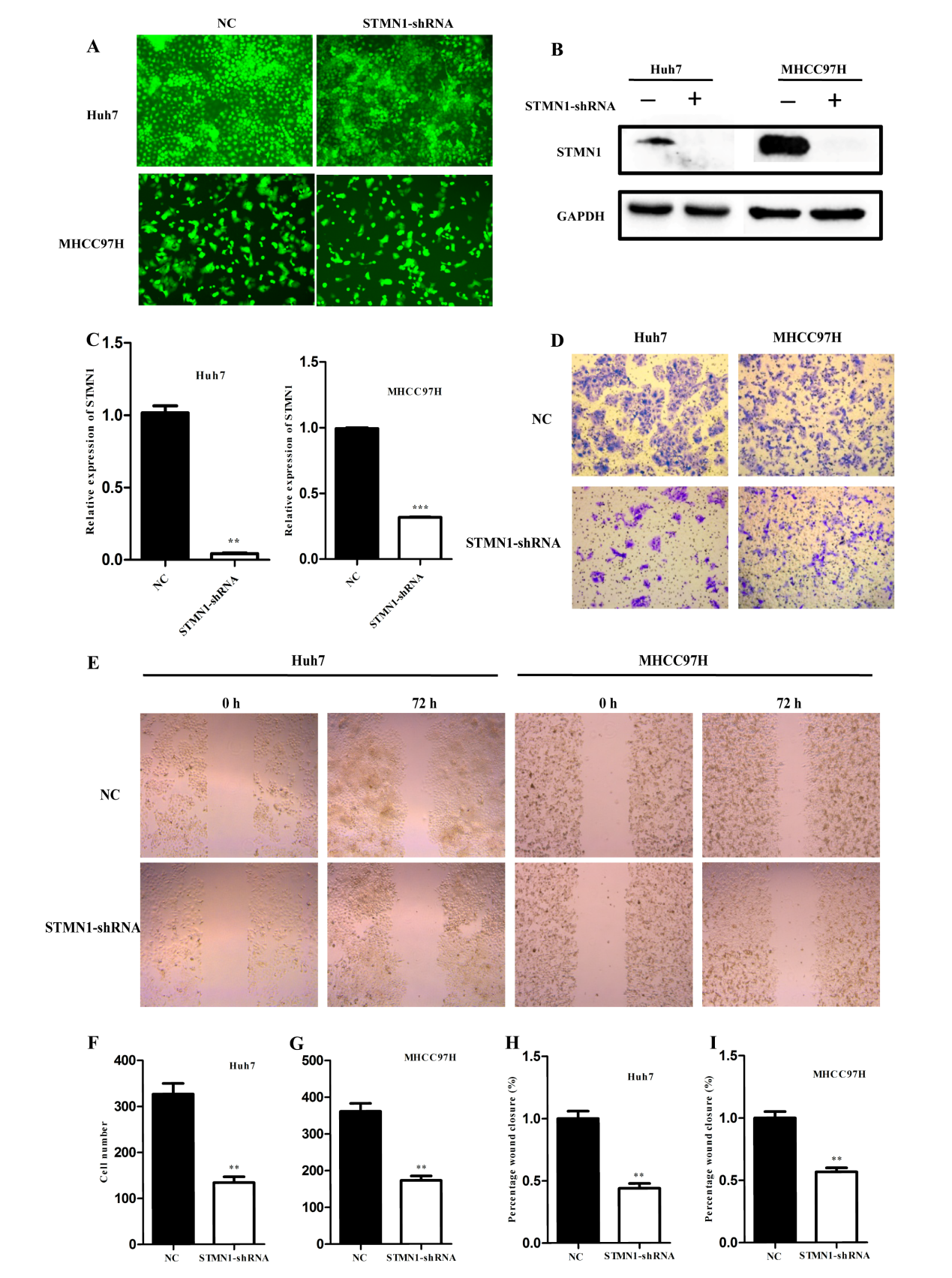


## Fig. S5. STMN1 knockdown inhibited the malignant phenotype of HCC cells in vitro.

(A, B, C) Confirmation of STMN1 knockdown (STMN1-shRNA) in Huh7 and MHCC97H cell lines by real-time QPCR. (D) STMN1 knockdown suppressed cell invasion in the transwell invasion assay. (E) STMN1 knockdown suppressed cell migration in the wound healing assay. (F, G) Quantification of invasive cells in the NC or STMN1-shRNA groups in the transwell invasion assay. (H, I) Quantification of migration cells in the NC or STMN1-shRNA groups in the wound healing assay.


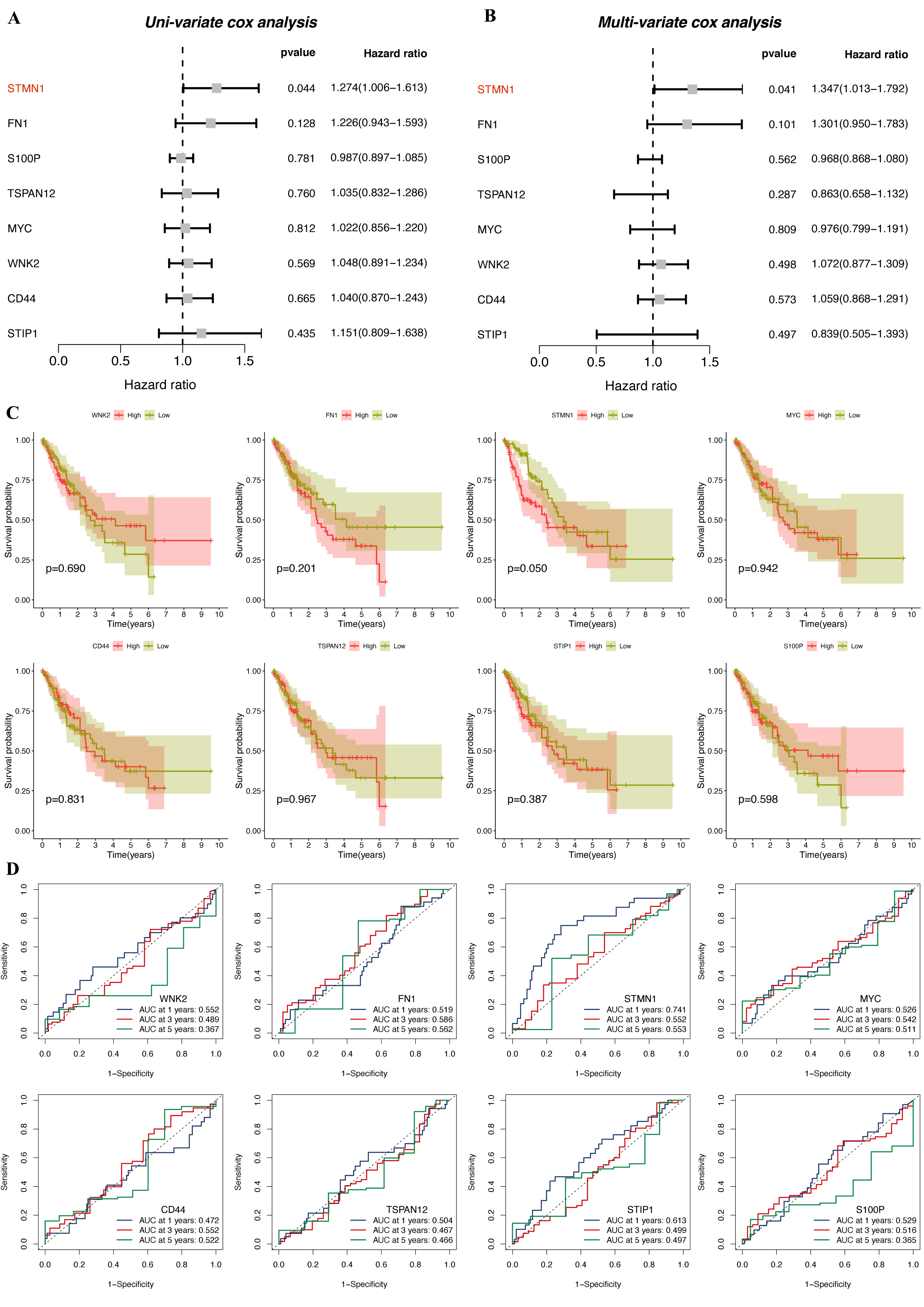


**Fig. S6. The comparative analysis of reported MVI-related biomarkers and STMN1 in early HCC using TCGA database**

(A, B) The uni- and multi-variate cox regression analyses of reported MVI-related biomarkers and STMN1. (C) Kaplan-Meier curves of recurrence displayed the correlation between clinical outcome and risk classification stratified by MVI-related biomarkers. (D) ROC analyses of reported MVI-related biomarkers and STMN1 in early HCC patients.


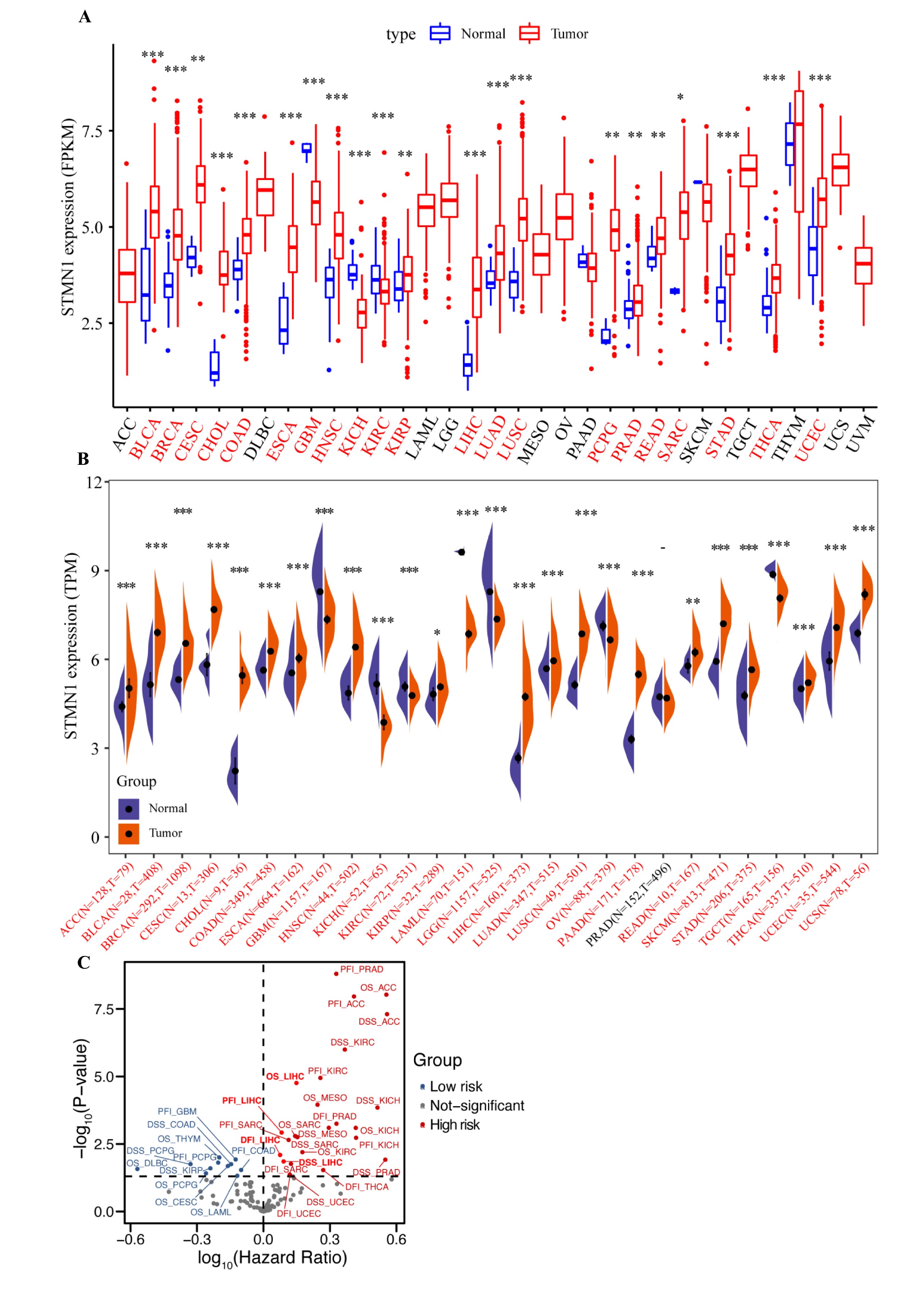


## Fig. S7. Differential expression analysis of STMN1 through the established databases for various cancers.

(A) Differential expression analysis of STMN1 only in TCGA database. Several cancers fail to run the analysis due to the lack of normal tissues. (B) Differential expression analysis of STMN1 in TCGA database combined with GTEx database. (C) Volcano plot of univariate cox analysis of STMN1 in 33 cancer types in TCGA database. The abbreviations used in the plot are as followed: OS, overall survival; DSS, disease specific survival; PFI, progression free interval; DFI, disease free interval.

##
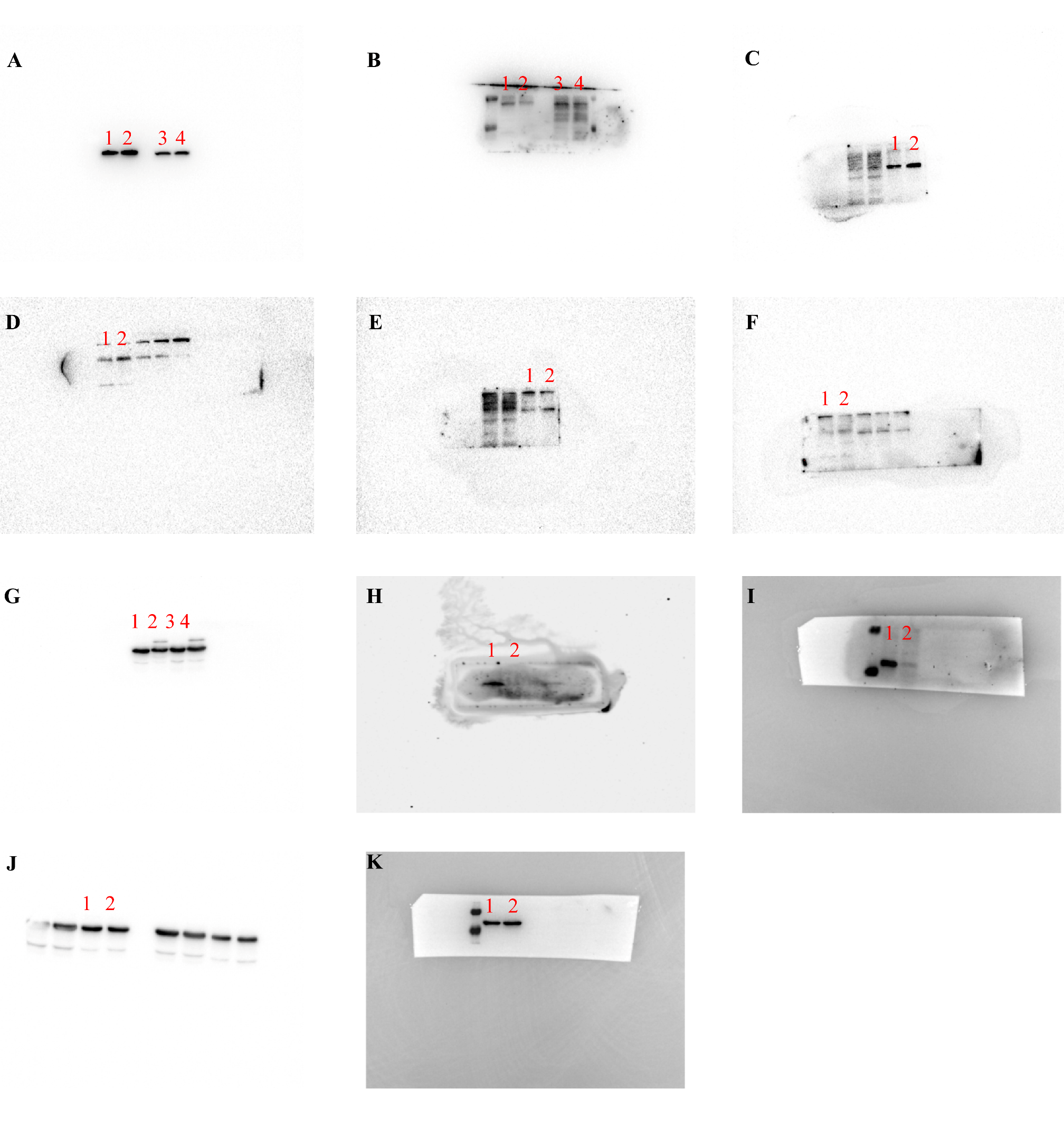
Fig. S8. The original western blots

(A) Lane 1: the original western blots of Ac-a-tubulin in Huh7 STMN1-shRNA (-) group; Lane 2: the original western blots of Ac-a-tubulin in Huh7 STMN1-shRNA (+) group; Lane 3: the original western blots of Ac-a-tubulin in MHCC97H STMN1-shRNA (-) group. Lane 4: the original western blots of Ac-a-tubulin in MHCC97H STMN1-shRNA (+) group. (B) Lane 1: the original western blots of pY397-FAK in Huh7 STMN1-shRNA (-) group; Lane 2: the original western blots of pY397-FAK in Huh7 STMN1-shRNA (+) group; Lane 3: the original western blots of pY397-FAK in MHCC97H STMN1-shRNA (-) group. Lane 4: the original western blots of pY397-FAK in MHCC97H STMN1-shRNA (+) group. (C) Lane 1: the original western blots of E-cadherin in Huh7 STMN1-shRNA (-) group; Lane 2: the original western blots of E-cadherin in Huh7 STMN1-shRNA (+) group. (D) Lane 1: the original western blots of E-cadherin in MHCC97H STMN1-shRNA (-) group; Lane 2: the original western blots of E-cadherin in MHCC97H STMN1-shRNA (+) group. (E) Lane 1: the original western blots of N-cadherin in Huh7 STMN1-shRNA (-) group; Lane 2: the original western blots of N-cadherin in Huh7 STMN1-shRNA (+) group. (F) Lane 1: the original western blots of N-cadherin in MHCC97H STMN1-shRNA (-) group; Lane 2: the original western blots of N-cadherin in MHCC97H STMN1-shRNA (+) group. (G) Lane 1: the original western blots of GAPDH in Huh7 STMN1-shRNA (-) group; Lane 2: the original western blots of GAPDH in Huh7 STMN1-shRNA (+) group; Lane 3: the original western blots of GAPDH in MHCC97H STMN1-shRNA (-) group. Lane 4: the original western blots of GAPDH in MHCC97H STMN1-shRNA (+) group. (H) Lane 1: the original western blots of STMN1 in Huh7 STMN1-shRNA (-) group; Lane 2: the original western blots of STMN1 in Huh7 STMN1-shRNA (+) group. (I) Lane 1: the original western blots of STMN1 in MHCC97H STMN1-shRNA (-) group; Lane 2: the original western blots of STMN1 in MHCC97H STMN1-shRNA (+) group. (J) Lane 1: the original western blots of GAPDH in Huh7 STMN1-shRNA (-) group; Lane 2: the original western blots of GAPDH in Huh7 STMN1-shRNA (+) group. (K)Lane 1: the original western blots of GAPDH in MHCC97H STMN1-shRNA (-) group; Lane 2: the original western blots of GAPDH in MHCC97H STMN1-shRNA (+) group. A-G corresponds to Figure 6A, H-K corresponds to Supplementary Fig. S5C.

## Supplementary Tables

**Supplementary Table 1: Clinicopathological characteristic of 130 early HCC patients**

| Variable | M0 | M1 | M2 |
| --- | --- | --- | --- |
| All cases | 51 | 42 | 37 |
| Age(year), ≥ 60: < 60 | 13: 38 | 20: 22 | 9: 28 |
| Gender, male: female | 43: 8 | 34: 8 | 26: 11 |
| HBe antigen  positive: negative | 13: 38 | 9: 33 | 8: 29 |
| HBs antigen positive: negative | 44: 7 | 33: 9 | 34: 3 |
| Liver fibrosis,Yes: No | 32: 19 | 28: 14 | 26: 11 |
| G1-2: G3 | 30: 2 | 24: 4 | 23: 3 |
| S1-2: S3-4 | 19: 13 | 14: 14 | 16: 10 |
| Liver cirrhosis, with: without | 17: 34 | 12: 30 | 9: 28 |
| Serum albumin (g/L),  > 40: ≤ 40 | 39: 12 | 30: 12 | 30: 7 |
| Serum bilirubin (umol/L), > 17: ≤ 17 | 17: 34 | 17: 25 | 11: 26 |
| ALT (U/L), > 40: ≤ 40 | 17: 34 | 10: 32 | 11: 26 |
| AST (U/L), > 40: ≤ 40 | 11: 40 | 4: 38 | 6: 31 |
| AFP (ug/L), ≥ 400: < 400 | 6: 45 | 14: 28 | 20: 17 |
| Tumor size (cm), ≥ 2: < 2 | 39: 12 | 34: 8 | 33: 4 |
| Cell differentiation,  III +Ⅳ: I + II | 37: 14 | 39: 3 | 36: 1 |
| Encapsulation,  No or Uncomplete: Complete | 37: 14 | 35: 7 | 34: 3 |

## Supplementary Table 2. Information of the datasets in present work.

| Datasets | Year | Platform | Counts of Samples used | Citation(s)(PMID) |
| --- | --- | --- | --- | --- |
| GSE10186 | 2009 | GPL5474 | 45 (Tumor)  17 (Tumor with MVI) | 19723656(1)  28644127(2) |
| GSE77509 | 2017 | GPL16791 | 20 (normal)  20 (Tumor)  20 (PVTT) | 28194035(3) |
| TCGA-LIHC | Data Release 18.0-July 08, 2019 | HTSeq-FPKM | 50 (normal)  206 (none-VI)  92 (Micro-VI)  16 (Macro-VI) | http://cancergenome.nih.gov |

Note: GSE, Gene Expression Omnibus Series; GPL, Gene Expression Omnibus Platform; LIHC, Liver Hepatocellular Carcinoma (HCC

**Supplementary Table 3. Primary antibodies used in the IHC and western blot assay.**

| Primary Antibody | Brand | Cat. No. |
| --- | --- | --- |
| Stathmin 1 | Cell Sigaling Technology | 3352s |
| CD34 | MXB Biotechnlogies | Kit-0004 |
| E-caderin | BD pharmingen | 610181 |
| N-caderin | Proteintech | 60335-1-1g |
| Anti-FAK (phospho Y397) | Abcam | ab81298 |
| Anti-alpha Tubulin (acetyl K40) | Abcam | ab24610 |

## References

1. Hoshida Y, Nijman SM, Kobayashi M, Chan JA, Brunet JP, Chiang DY*, et al.* Integrative transcriptome analysis reveals common molecular subclasses of human hepatocellular carcinoma. *Cancer Res* **69,** 7385-7392 (2009)

2. Shtraizent N, DeRossi C, Nayar S, Sachidanandam R, Katz LS, Prince A*, et al.* MPI depletion enhances O-GlcNAcylation of p53 and suppresses the Warburg effect. *Elife* **6,** (2017)

3. Yang Y, Chen L, Gu J, Zhang H, Yuan J, Lian Q*, et al.* Recurrently deregulated lncRNAs in hepatocellular carcinoma. *Nat Commun* **8,** 14421 (2017)
